# Child t-shirt size data set from 3D body scanner anthropometric measurements and a questionnaire

**DOI:** 10.1016/j.dib.2017.02.025

**Published:** 2017-02-16

**Authors:** A. Pierola, I. Epifanio, S. Alemany

**Affiliations:** a Biomechanics Institute of Valencia, Universidad Politécnica de Valencia, Valencia 46022, Spain; b Dept. Matemàtiques and Institut de Matemàtiques i Aplicacions de Castelló, Universitat Jaume I, Castelló 12071, Spain

**Keywords:** Anthropometry, Ergonomics, Clothing fit, Classification

## Abstract

A dataset of a fit assessment study in children is presented. Anthropometric measurements of 113 children were obtained using a 3D body scanner. Children tested a t-shirt of different sizes and a different model for boys and girls, and their fit was assessed by an expert. This expert labeled the fit as 0 (correct), −1 (if the garment was small for that child), or 1 (if the garment was large for that child) in an ordered factor called Size-fit. Moreover, the fit was numerically assessed from 1 (very poor fit) to 10 (perfect fit) in a variable called Expert evaluation. This data set contains the differences between the reference mannequin of the evaluated size and the child׳s anthropometric measurements for 27 variables. Besides these variables, in the data set, we can also find the gender, the size evaluated, and the size recommended by the expert, including if an intermediate, but nonexistent size between two consecutive sizes would have been the right size. In total, there are 232 observations. The analysis of these data can be found in Pierola et al. (2016) [2].

**Specifications table**

**Table:** 

Subject area	*Engineering*
More specific subject area	*Anthropometry, Ergonomics, Clothing fit*
Type of data	*Text file*
How data was acquired	*A Vitus Smart 3D body scanner from Human Solutions was used. The expert that assessed the fit was an anthropometry expert technician with a degree on pattern making.*
Data format	*Table in Data format*
Experimental factors	*The child mannequins MNQ 0–12 from ASEPRI(Spanish Association of Children׳s Products) were scanned and measured for comparing the mannequin and child body dimensions.*
Experimental features	*The number of children aged between 3 and 12 years participating in the experimental study was balanced according to age ranges (3–4, 4–6, 6–8, 8–10, 10–12 years), with an equivalent number of boys and girls.*
Data source location	*Spain*
Data accessibility	*The dataset is available in this article*

**Value of the data**

• To the best of our knowledge, this is the first data set about the garment matching problem in children.
• The data can be used to benchmark and compare classifiers in ordinal classification problems.
• The output is multivariate: an ordinal factor (Size-fit) and a numeric variable (Expert evaluation). The data set can also be used to benchmark this kind of data in a real problem.
• The data set can serve to benchmark classifiers when uncertainties are present. In the classical supervised classification paradigm it is usually assumed that the definition of the classes is made objectively, without arbitrariness or uncertainty [1], but this is not the case. In this problem, the class definition is more quantitative than qualitative. Moreover, it could happen that none of the sizes fits the child well, or two sizes could be right sizes.
• Data come from a real and important problem.

## 1 Data

Several observations in the data set are generated by each child. For each of the sizes that have been assessed on the child, an observation (a line in the data set) is generated. Observations consist of the differences between the reference mannequin of the evaluated size measurements and the child׳s anthropometric measurements, the tested size and the assessment process results. These results consist of the size which best fits the child according to the pattern making expert׳s criteria, if any (it could happen that none of the sizes fitted the child well). This expert could chose only one size as the right one for the child. The right size was labeled as 0. The rest of sizes evaluated were labeled −1 or 1 depending on whether the t-shirt was smaller or larger. This corresponds to the Size-fit variable. The Int-size variable indicates if an intermediate, but inexistent size between two consecutive sizes would have been the correct size. Moreover, the expert assessed the fit with a number between 1 and 10, where 1 means a very poor fit and 10 a perfect fit, and 6 a normal fit. This corresponds to the Expert evaluation variable*.*

Only integer numbers were used by the expert. Note that there is not analytic relationship between Expert evaluation and Size-fit variables*.*

## 2 Experimental design, materials and methods

During the fitting test, the t-shirts were tried on the children and a pattern making expert answered a questionnaire about his perception of the t-shirt fit. In the fit study, three sizes were evaluated for current use on each child: his/her supposed correct size, the immediately smaller size and the immediately larger size, if these were manufactured. Afterwards, the expert selected the size which best suited the child. Nevertheless, sometimes not all children tried on the three sizes, but only two sizes or even one, depending on their cooperation degree. The sizes are denoted as year 2, 3, 4, 5, 6, 8, 10 and 12*.*

The gender of the children was recorded. The children were scanned in a standing position with a Vitus Smart 3D body scanner from Human Solutions. The scanner is a non-intrusive laser system formed by four columns allocating the optic system. It moves from the head to the feet in ten seconds performing a sweep of the body. A head cap and tight underwear were worn by children for scanning. A total of 34 anthropometric measurements were estimated semi-automatically with digital tape measurement software, combining automatic measurements based on geometric characteristic points with a manual review. Furthermore, for making easier the measurement extraction, various physical markers were fixed during the scanning process and virtual landmarks were also determined on the children׳s scans. Note that we have discarded several variables of the whole set of 34 variables, such as ankle perimeter, since they do not have influence in the fitting of the t-shirt according to design experts. So, the data set include a total of 27 anthropometric variables, whose meaning can be seen in Table 1. Remember that these variables are the difference between the mannequin of the tested size measurements and the child׳s anthropometric measurements in millimeters. Table 2 shows the rest of the variables for each observation.The data set was analyzed in [2]. The R code for analyzing the data set as made in [2] can be found in http://www3.uji.es/~epifanio/RESEARCH/ensemble.rar.

Tables of body dimensions by size according to ASEPRI can be found in [3]. The collection of mannequins matches these measurements.

As regards the garment sizes, Table 3, Table 4 report the measurements that provide a good fit according to the brand׳s size chart for each size for boys and girls, respectively.

## Figures and Tables

**Table 1:** Anthropometric measurements in child t-shirt size data set.

***Code***	***Physical meaning***
*Stature*	*Body height*
*7CV_height*	*7 cervical vertebrae (CV) height*
*Mid_neck*	*Mid neck girth*
*Neck*	*Neck at base girth*
*Head*	*Head circumference*
*Shoulder_width*	*Horizontal shoulder width between acromia*
*Shoulder_length*	*Left shoulder length*
*Armpits_width*	*Width armpits*
*Bust_width*	*Bust points width*
*Neck_waist*	*Length neck-waist over chest*
*Bust_neck*	*Bust point to neck*
*Bust*	*Bust/chest girth (horizontal)*
*Back_width*	*Across back width (armpit level)*
*Neck_armpits*	*Length neck-armpits line*
*Neck_waist*	*Vertical length neck-waist*
*Crotch*	*Crotch length*
*Front_crotch*	*Front crotch length*
*Rear_crotch*	*Rear crotch length*
*Waist*	*Waist girth*
*Buttock*	*Buttock girth*
*Hip*	*Hip girth*
*Belly*	*Belly circumference*
*Arm_7CV*	*Arm length left to 7 CV*
*Arm_length*	*Arm length left*
*Upper_arm_length*	*Upper arm length left*
*Upper_arm_girth*	*Upper arm girth*
*Wrist*	*Wrist girth left*

**Table 2:** Variables related with the fit assessment in child t-shirt size data set.

***Name***	***Meaning***
*User code*	*The code of the child*
*Size_fit*	*Ordered factor. The expert labeled the fit as 0 (correct), -1 (if the garment was small for that child), or 1 (if the garment was large for that child)*
*Expert_evaluation*	*The fit was numerically assessed from 1 (very poor fit) to 10 (perfect fit)*
*Gender*	*Gender of the child: V (boy) and M (girl).*
*Size_evaluated*	*Factor with the size evaluated*
*Int_size_expert*	*It indicates if an intermediate, but nonexistent*
	*size between two consecutive sizes would have been the right size*
*Size_expert*	*Size recommended by the expert*

**Table 3:** Sizing table for boys in cm.

***Size***	***Y02***	***Y03***	***Y04***	***Y05***	***Y06***	***Y08***	***Y10***	***Y12***
*Stature*	*87–92*	*93–98*	*99–104*	*105–110*	*111–116*	*117–128*	*129–140*	*141–152*
*Bust girth*	*52*	*54*	*56*	*58*	*60*	*64*	*68*	*74*
*Waist girth*	*50*	*51.5*	*53*	*54.5*	*56*	*59*	*62*	*66*
*Hip girth*	*56*	*58.5*	*61*	*63.5*	*66*	*71*	*76*	*81*

**Table 4:** Sizing table for girls in cm.

***Size***	***Y02***	***Y03***	***Y04***	***Y05***	***Y06***	***Y08***	***Y10***	***Y12***
*Stature*	*87–92*	*93–98*	*99–104*	*105–110*	*111–116*	*117–128*	*129–140*	*141–152*
*Bust girth*	*52*	*54*	*56*	*58*	*60*	*64*	*68*	*73*
*Waist girth*	*50*	*51*	*52*	*53*	*54*	*56*	*59*	*62*
*Hip girth*	*56*	*58.5*	*61*	*63.5*	*66*	*71*	*76*	*81.5*
