# Supplementary material for: Child t-shirt size data set from 3D body scanner anthropometric measurements and a questionnaire
Source: Data Brief. 2017 Feb 16;11:311–5. doi: 10.1016/j.dib.2017.02.025 (PMC5328721; doi:10.1016/j.dib.2017.02.025)
Supplement: Supplementary file 1 — Supplementary material [file mmc1.docx]

We wish to confirm that there are no known conflicts of interest associated with this work.
